# Moral distress and injury in the public health professional workforce during the COVID-19 pandemic

**DOI:** 10.1093/pubmed/fdad010

**Published:** 2023-03-01

**Authors:** Steven M A Bow, Peter Schröder-Bäck, Dominic Norcliffe-Brown, James Wilson, Farhang Tahzib

**Affiliations:** Department of Philosophy, University College London, Gower Street, London WC1E 6BT, UK; Institute for Ethics and History, University of Applied Sciences for Police and Public Administration in North Rhine-Westphalia (HSPV NRW), Campus Aachen, Dennewartstrasse 25-27, 52068 Aachen, Germany; British Medical Association, BMA House, Tavistock Square, London WC1H 9JP, UK; Department of Philosophy, University College London, Gower Street, London WC1E 6BT, UK; Faculty of Public Health, 4 St Andrews Place, London NW1 4LB, UK

**Keywords:** ethics, management and policy, mental health

## Abstract

**Background:**

There is growing concern about moral distress and injury associated with the COVID-19 pandemic in healthcare professions. This study aimed to quantify the nature, frequency, severity and duration of the problem in the public health professional workforce.

**Methods:**

Between 14 December 2021 and 23 February 2022, Faculty of Public Health (FPH) members were surveyed about their experiences of moral distress before and during the pandemic.

**Results:**

In total, 629 FPH members responded, of which, 405 (64%; 95% confidence interval [95%CI] = 61–68%) reported one or more experience of moral distress associated with their own action (or inaction), and 163 (26%; 95%CI = 23–29%) reported experiencing moral distress associated with a colleague’s or organization’s action (or inaction) since the start of the pandemic. The majority reported moral distress being more frequent during the pandemic and that the effects endured for over a week. In total, 56 respondents (9% of total sample, 14% of those with moral distress), reported moral injury severe enough to require time off work and/or therapeutic help.

**Conclusions:**

Moral distress and injury are significant problems in the UK public health professional workforce, exacerbated by the COVID-19 pandemic. There is urgent need to understand the causes and potential options for its prevention, amelioration and care.

## Introduction

The increasing academic interest in the phenomena of moral distress and moral injury has been heightened by concerns about the impact of the COVID-19 pandemic—with its parallel threats of physical and mental harms to those involved in, and affected by, the pandemic response.^1–3^

‘Moral distress’ refers to the psychological distress precipitated by the experience of an incident to which the subject attaches a significant moral judgement, i.e. a ‘moral event’.^4^ ‘Moral injury’ refers to the long term and severe negative effects of such a moral event, typically requiring professional therapy, and, as such, can be seen as either a type, or a potential consequence, of moral distress.^5^ It should be noted that there remains debate about the appropriate definition of both moral distress and moral injury.^4–11^

Experiences of moral distress and injury are a concern because of both their causes and their effects. Firstly, the incidents that precipitated the moral distress and injury may represent injustices in the world that need to be addressed for their own sake. Secondly, studies have implicated moral distress and injury in a range of physical and mental harms to staff, and consequent workforce losses, deterioration in the quality of care and poorer outcomes for patients and their families.^12^^,^^13^ These direct and indirect harms need to be prevented, mitigated or treated.

The majority of research on moral distress and injury has focused on healthcare and military professionals, respectively.^14^^,^^15^ However, anyone with the capacity to evaluate their own actions can experience moral distress and injury, and there has been growing interest in investigating these experiences in other fields.

To date, there have been a few studies of moral distress and injury in the public health professional workforce. One qualitative study of Australian and Canadian health promotion practitioners retrospectively identified moral distress themes from interview transcripts, and an international survey of field epidemiologists found that 91% reported work-related moral distress, with 26% experiencing it ‘frequently’ or ‘always’.^16^^,^^17^

Both studies assumed the most narrow definition of moral distress, i.e. distress that arises because external constraints prevent the subject acting in accordance with their moral judgement, and so do not provide insight into the full range of distress arising from public health professionals’ moral judgements in the workplace.

In addition, neither study focused on UK public health professionals, and both predated the COVID-19 pandemic. In contrast, recent membership surveys by the UK doctors’ and medical students’ trade union and professional body, the British Medical Association (BMA), and the UK public health professional membership body, the Faculty of Public Health (FPH) hinted that, in the wake of the second wave of the COVID-19 pandemic, moral distress and injury could be a significant problem in this cohort.

The BMA surveyed UK doctors between March and April 2021 about their experiences of moral distress and injury.^18^ The majority of respondents (78.4%) said ‘moral distress’ resonated with their experiences at work, of whom 86.2% and 70.8%, respectively, said they had experienced moral distress in relation to care provided by themselves, or their colleagues (which equates to 63% and 51% of all respondents, respectively). Almost all (96.4%) said the pandemic had made moral distress more likely, and almost a fifth (19.5%) stated ‘Public health decisions’ were a factor. However, out of 1933 respondents, only 2.1% (⁓40 respondents) were public health specialists, and non-medical public health specialists were outside the survey’s scope.

The FPH surveyed its members (UK public health professionals, including medical doctors and colleagues with other backgrounds) about their physical and mental wellbeing between April and May 2021, finding that the pandemic had taken a toll on respondents’ mental wellbeing.^19^ It reported a key theme was being ‘morally compromised, having to give advice they thought was wrong’, although it did not specifically ask about moral distress or report any further detail about its nature.

The paucity of research into moral distress and injury in public health is surprising, since public health professionals routinely face morally ambiguous and challenging situations, and their decisions were uniquely under the spotlight during the pandemic.

To address this knowledge gap, the FPH conducted a survey of its members, focusing specifically on the collation of reports of moral distress and injury since the start of the COVID-19 pandemic. This aimed to assess the nature, frequency, severity, duration and impact of moral distress encountered, and its association with individual characteristics and ethical training.

## Methodology

### Design

This cross-sectional survey was aimed at the professional members of the UK public health workforce. An invitation was emailed to all FPH members (c. 4000)^20^ and data were collected electronically via SurveyMonkey between 14 December 2021 and 23 February 2022. A minimum sample size of 384 was calculated to give precision within a 5% margin of error with 95% confidence.

### Measures

We adapted existing tools for characterizing moral distress and injury in clinical and military personnel^21^^,^^22^ to make them relevant to the public health context. Interpreting the concepts of moral distress and injury in their broadest sense, we asked three groups of questions distinguishing between the agent’s evaluation of their action or decision (knowing it was wrong, knowing it was right or uncertain), and a further question group about the actions of third parties (see supplementary information for survey questionnaire). Throughout the survey we used the terminology of ‘ethical/ethically problematic’ and ‘morally right/wrong’ in parallel, to indicate that we were treating the two phrases as being synonymous, precisely because we were aware that some hold that ‘morality’ and ‘ethics’ are not synonyms, but refer to subjective and objective judgements respectively, and to avoid this creating unnecessary ambiguity in the questionnaire.

Within each of these question groups we asked whether the respondent had experienced that type of distress, its absolute frequency and intensity (rated on scale similar to that used in the Moral Distress Scale Revised: MDS-R), its frequency relative to the year before the pandemic, its duration, and, as a proxy measure of moral injury, whether it led to time off work or seeking therapeutic help.^21^ The MDS-R used a 0–4 scale; ours used 1–5, since a score of ‘0’ might imply no experience of moral distress, and only those who had already indicated having experienced moral distress were asked the question. We also asked respondents open-ended questions about the perceived causes of moral distress and potential mitigating actions.

The primary outcome measures were the reported experiences of moral distress of any kind, associated with (i) the agent’s own action (or inaction) and (ii) a colleague’s or organization’s action (or inaction).

In order to classify respondents, we included questions about demographics, professional background and ethical knowledge and training.

### Analysis

Confidence intervals were calculated for moral distress and injury outcomes using the Wilson score method. Bivariate analysis was used to test the associations between respondent characteristics and ethical training levels and reported experience of moral distress. Not stated and missing values were excluded, and the ‘other’ gender category was excluded due to small numbers. Logistic regression analyses were used to explore which associations remained when adjusted for variation in other characteristics.

### Ethics

No formal ethical approval was required; staff were recruited by virtue of their professional role.^23^ Consent was obtained at the survey outset. No personal data were collected. Respondents were advised not to include real names or details of specific workplaces in their responses.

## Results

### Respondents

In total, 629 survey responses were received. Table 1 shows a summary of demographic and professional characteristics of respondents (see Supplementary Table 1 for detailed breakdown).

**Table 1:** Summary of demographic and professional characteristics of respondents

	Respondents			Respondents	
	N	%		N	%
**Gender**			**Country**		
Female	308	49	England	479	76
Male	148	24	UK devolved administration	87	14
Other	3	0.5	Outside the UK	63	10
Unknown^a^	170	27	**Employer**		
**Age**			Local government	206	33
25–34	55	9	National government/agency	181	29
35–44	115	18	Academic institution	76	12
45–54	112	18	Healthcare	53	8
55–64	104	17	Other	86	14
65+	67	11	Not stated/not applicable	27	4
Unknown ^a^	176	28	**Job role**		
**Disability**			Consultant/Specialist	207	33
Yes	49	8	Public Health Trainee	132	21
No	408	65	Public Health Academic	59	9
Unknown ^a^	172	27	Director of Public Health	49	8
**Religion**			Retired	39	6
No religion	231	37	Other	141	22
Christian	148	24	Not stated	2	0.3
Other religion	41	7	**Public health career length**		
Unknown ^a^	209	33	5 years or less	147	23
**Ethnicity**			6–10 years	112	18
White	375	60	11–15 years	80	13
Other	70	11	16–20 years	78	12
Unknown ^a^	184	29	˃20 years	212	34
			**Clinically trained**		
			Yes	369	59
			No	259	41
			Not stated	1	0.2

a Includes those who responded ‘Prefer not to say’ and those who did not provide any response to the question.

More respondents reported their gender as female (49%) than male (24%; note that over a quarter of respondents did not provide full demographic information), all respondents were aged 25 years or older, 8% reported a disability. Over a third (37%) reported having no religion, about a quarter (24%) were Christian and 7% followed another religion, and 60% reported being white.

In total, 76% reported working in England, with 14% working in a UK devolved administration. A third (33%) worked for local government, 29% in a national public health agency or government department, with others working in academia (12%), healthcare (8%) or other settings (14%).

A third were public health consultants/specialists, 21% were trainees/registrars, 9% were academics, 8% were directors of public health and 6% were retired. There were responses from people at every point in their career ranging from those with five or fewer years of public health work experience (23%), to over 20 years (34%). In total, 59% were clinically trained.

The number of responses received equates to a response rate of ⁓16% of the FPH membership,^20^ which was high compared to the BMA survey (⁓1.2% of its 162 346 members responded).^18^^,^^24^ In total, ⁓38% of all directors of public health and public health trainees, 20% of public health academics and 16% of public health consultants responded.^25^ When missing data were excluded, the pattern of respondents’ characteristics were broadly representative of the public health workforce.^26^

Tables 2 and 3 show the respondents’ reported level of ethical training and preparedness. A substantial minority reported having heard the terms ‘moral distress’ and ‘moral injury’ before (46% and 38%, respectively). About a third (36%) had received some ethical training as part of their formal education and about a quarter (26%) had since then. About a quarter (24%) said more such training would have helped them to handle the situation in which they reported experiencing moral distress. On a 0–4 scale, respondents reported greater confidence that they could identify if a situation contained an ethical dilemma (mean = 2.9), than that they knew what principles, tools or frameworks for help making a decision when confronted with ethical issues (mean = 2.2), or that they or colleagues were adequately prepared to face ethical issues (mean = 2.3 and 1.8, respectively).

**Table 2:** Respondents’ awareness of terms and ethical training received or required

	Respondents			Respondents	
	N	%		N	%
**Awareness of term**	**Moral distress**		**Moral injury**		
Yes	288	46	238	38	
No/not sure	190	30	239	38	
Not stated	151	24	152	24	
**Ethical training received**	***During* formal education**		***Since* formal education**		
Yes	225	36	164	26	
No/not sure	252	40	312	50	
Not stated	152	24	153	24	
**More ethical training would have helped reduce moral distress and injury**					
Yes	150	24			
No/not sure	252	40			
Not applicable	72	11			
Not stated	155	25			

**Table 3:** Respondents’ reported level of ethical preparedness

	N	%	N	%	N	%	N	%
	Can easily identify ethical dilemmas		Know what principles etc. to use for ethical issues		Adequately prepared to face ethical issues		Colleagues are adequately prepared to face the ethical issues	
0 (Never)	0	0	21	3	10	2	14	2
1	18	3	112	18	90	14	144	23
2	90	14	131	21	161	26	229	36
3	299	48	179	28	173	28	71	11
4 (Always)	67	11	31	5	37	6	12	2
Not stated	155	25	155	25	158	25	159	25
*Mean*	*2.9*		*2.2*		*2.3*		*1.8*	

### Responses

In total, 405 (64%; 95% confidence interval [95%CI] = 61–68%) respondents reported having one or more experience of moral distress associated with their own action (or inaction) since the start of the pandemic. This is broken down in Table 4. Distress arising from doing something they judged to be ethically problematic or morally wrong was reported by 52% (95%CI = 48–55%), from doing something they judged morally right, by 38% (95%CI = 35–42%) and from doing something about which they were morally uncertain, by 24% (95%CI = 23–29%). Additionally, 163 (26%; 95%CI = 23–29%) reported experiencing moral distress associated with a colleague’s or organization’s action (or inaction).

**Table 4:** Experiences of types of moral distress since the start of the pandemic by survey respondents

	Agent’s moral evaluation of action									Due to colleague’s/ organization’s actions		
	Ethically problematic/morally wrong			Ethical/morally right			Uncertain					
	N	%	95% CIs	N	%	95% CIs	N	%	95% CIs	N	%	95% CIs
**Experienced distress since start of pandemic**												
Yes	324	52	48–55	241	38	35–42	152	24	21–28	163	26	23–29
No/not sure	283	45	41–49	309	49	45–53	360	57	53–61	330	52	49–56
Not stated	22	3	2.3–5.2	79	13	10–15	117	19	16–22	136	22	19–25
**Frequency**												
1 (Rarely)	24	7	5.0–11	30	12	8.9–17	15	10	6.1–16	27	17	12–23
2	61	19	15–23	53	22	17–28	25	16	11–23	32	20	14–26
3	80	25	20–30	70	29	24–35	44	29	22–37	46	28	22–36
4	52	16	12–20	37	15	11–20	38	25	19–32	29	18	13–24
5 (Very frequently)	27	8	5.8–12	29	12	8.5–17	22	14	10–21	27	17	12–23
Not stated	80	25	20–30	22	9	6.1–13	8	5	2.7–10	2	1	0.3–4.4
Mean	3.0			2.9			3.2			3.0		
**Frequency relative to year before pandemic**												
Less often	47	15	11–19	40	17	12–22	24	16	11–22	24	15	10–21
About the same	3	1	0.3–2.7	28	12	8·.2–16	12	8	4.6–13	22	13	9.1–20
More often	181	56	50–61	141	59	52–65	99	65	57–72	105	64	57–71
Not sure	2	1	0.2–2.2	1	0.4	0.1–2.3	2	1	0.4–4.7	3	2	0.6–5.3
Not applicable	13	4	2.4–6.7	8	3	1.7–6.4	6	4	1.8–8·3	5	3	1.3–7.0
Not stated	78	24	20–29	23	10	6.4–14	9	6	3.1–11	4	2	1.0–6.1
**Level of distress**												
1 (A little)	8	2	1.3–4.8	13	5	3.2–9.0	10	7	3.6–12	5	3	1.3–7.0
2	22	7	4.5–10	30	12	8.9–17	25	16	11–23	19	12	7.6–17
3	64	20	16–24	63	26	21–32	44	29	22–37	52	32	25–39
4	75	23	19–28	56	23	18–29	35	23	17–30	49	30	24–37
5 (Great extent)	66	20	16–25	51	21	16–27	24	16	11–22	27	17	12–23
Not stated	89	27	23–33	28	12	8.2–16	14	9	5.6–15	11	7	3.8–12
Mean	3.7			3.5			3.3			3.5		
**Duration**												
Less than a day	17	5	3.3–8.2	26	11	7.5–15	12	8	4.6–13	15	9	5.7–15
Between a day and a week	62	19	15–24	61	25	20–31	46	30	24–38	36	22	16–29
Between a week and month	54	17	13–21	53	22	17–28	32	21	15–28	35	21	16–28
More than a month	111	34	29–40	77	32	26–38	51	34	27–41	70	43	36–51
Not stated	80	25	20–30	24	10	6.8–14	11	7	4.1–12	7	4	2.1–8.6
**Time off work/sought therapeutic help**												
Yes	36	11	8.1–15	37	15	11–20	15	10	6.1–16	24	15	10–21
No	209	65	59–70	175	73	67–78	123	81	74–86	131	80	74–86
Not stated	79	24	20–29	29	12	8.5–17	14	9	5.6–15	8	5	2.5–9.4

#### Frequency

On a scale from 1 (rarely) to 5 (very frequently), those who reported experiencing distress reported a mean rating of 3.0, 2.9, 3.2 and 3.0 distress arising from one’s own actions that were morally wrong, morally right, morally uncertain and other actions, respectively. Across all types of moral distress, the majority reported that the experience occurred more frequently than in the 12 months before the pandemic (range = 56–65%).

#### Level of distress

On a scale from 1 (a little) to 5 (great extent), those who reported experiencing distress reported a mean rating of 3.7, 3.5, 3.3 and 3.5 distress arising from one’s own actions that were morally wrong, morally right, morally uncertain and other actions, respectively. Over a fifth of those reporting distress arising from their own actions that they judged were certainly morally wrong or morally right said they experienced distress to a ‘great extent’ (20% [95%CI = 16–25%] and 21% [95%CI = 16–27%], respectively).

#### Duration

Across all types of moral distress, over a half of those who experienced it said it continued for more than a week (range = 51–64%), and around a third said it continued more than a month (range = 32–43%).

#### Moral injury

In total, 56 respondents reported that they had to take time off work and/or seek therapeutic help due to moral distress. This equated to 9% (95%CI = 7–11%) of the total sample, and 14% (95%CI = 11–18%) of those who reported experiencing moral distress of some kind.

#### Associations between respondent characteristics and moral distress

Logistic regression analysis found that the odds of experiencing moral distress were positively associated with white ethnicity, career lengths of 6–10 years, and disability, and inversely associated with age, public health trainee and ‘other’ job roles, and post-formal education ethical training. Bivariate associations and the results of logistic regression results are reported fully in supplementary materials (Supplementary Tables 2 and 3).

## Discussion

### Main finding of this study

A majority of respondents (64%, *n* = 405) reported having experienced moral distress associated with their own action (or inaction), and over a quarter (26%, *n* = 163) reported experiencing moral distress associated with a colleague’s or organization’s action (or inaction) since the start of the pandemic. The majority (range = 56–65%) reported that the experience occurred more frequently than in the 12 months before the pandemic and that the moral distress lasted more than a week (range = 51–64%). In 56 respondents (9% of total sample, 14% of those with moral distress), this moral distress led to moral injury (in terms of having to take time off work and/or seek therapeutic help).

### What is already known on this topic

The proportion of respondents in our survey who reported moral distress associated with their own actions (64%) was similar to proportion of doctors in the BMA survey who reported moral distress associated with their ability to provide care (63%).^18^

However, when it came to moral distress associated with the actions and omissions of third parties, the proportion was lower in our survey (26%) compared with doctors in the BMA survey (51%). This is somewhat surprising, especially since the question used in our survey was broader, asking about moral distress associated with actions by organization’s, not just other colleagues, which would have been expected to capture more incidents, all things being equal. This could be an artefact of differences between the two surveys, e.g. in their timing and sampling methods. Alternatively, it could reflect a real difference between the nature and experiences of moral distress and injury in clinical and public health professionals, which would lend justification to calls for further research in the latter. That said, reports of ‘third-party moral distress’ rely on individuals observing and judging the actions and intentions of others, which may make them less a reliable measure of actual events.

### What this study adds

The 405 reports of moral distress we received are likely to represent just the tip of the iceberg of moral distress in the public health professional workforce associated with the pandemic. The study confirms that moral distress is a significant problem for UK public health professionals, has been exacerbated by the pandemic, and has the potential to cause harm to individual professionals, the workforce as a collective, and, consequently, the populations they serve.

The identification of some moral distress in the workforce may not, in itself, be considered surprising. An absence of moral distress could only suggest, on the one hand, the implausible idea that, in public health, there is never any doubt, disagreement or difficulty in decision-making, or on the other, that public health professionals are entirely incapable of experiencing (or at least detecting and articulating) moral thoughts and feelings. To that degree, our results are reassuring, insofar as they indicate that the moral faculties of the UK public health professional workforce are alive and well. However, to the extent that the results indicate the presence, and increase in frequency, of morally troubling incidents since the start of the pandemic, they are cause for concern and reflection.

The reasons for concern are 2-fold. First, because of the potential negative consequences of moral distress and injury (regardless of their cause) on the health and wellbeing of public health professionals, the cumulative impact on the public health professional workforce, and the indirect impact on the public they serve. Second, because these morally troubling incidents may reflect serious deficiencies in the public health workforce, institutions or wider system. This is not a necessary conclusion—moral distress may be an inescapable element of working in public health, due to the inevitable limitations on what action is feasible due to the complexity of the decision-making context, and unavoidable resource constraints. However, the possibility that it is a signal of major systemic failure must be taken seriously. This has implications for policymaking about the need to safeguard the public health workforce on the one hand, and to evaluate the condition of the wider public health system, on the other.

Further work is needed in order to understand the respondents’ perceived causes, and proposed ways to address the moral distress and injury they have suffered, as well as to explore the role of ethical training and education on these outcomes.

### Limitations of this study

The response rate was too low to assume a representative sample. Indeed, it seems likely that individuals who had experience moral distress and injury during the pandemic, and/or had *a priori* familiarity with the concepts of moral distress and injury, would be more likely to take the time to respond to an email invitation to a survey on the topic, thus leading to selection bias. The survey asked about experiences since the COVID-19 pandemic, so findings may not generalize to non-pandemic public health practice. There is a lack of validated measures for moral distress and injury in the public health discipline, and further work is needed to develop a robust instrument for measuring moral distress and injury in public health professionals. We used discrete categories for employer and job role, which ignored the possibility that individuals may have worked in multiple roles during the pandemic period, although we addressed this by asking respondents to report where they ‘primarily’ worked, and what ‘best describes’ them, in order to reduce the risk of systematic misclassification. There was a drop-off in response to questions located towards the end of the survey. Almost all respondents answered the question about the first type of moral distress, and only 78% answering about the fourth. About a quarter of respondents did not answer the later questions about demographics, and ethical knowledge and training. Due to the cross-sectional study design, it was not possible to infer causal relationships from associations between moral distress and respondent characteristics.

## Conclusion

Our findings, taken together with the existing literature, demonstrate that moral distress and injury are indeed significant problems in the UK public health professional workforce, and have been exacerbated by the COVID-19 pandemic. This is concerning both as a possible indicator of injustices perpetrated during the pandemic, and as a threat to the wellbeing of the workforce, and thus demands urgent attention. It has major policy implications, though further work is needed to understand the specific causes of and potential solutions to this moral distress, and the possible role of ethical training in preventing and mitigating its impact.

## Supplementary Material

FPH_Moral_distress_data_paper-JPH_v1-SUPP_fdad010

## Data Availability

All aggregate survey data is provided in the manuscript or supplementary information. Record-level data cannot be shared, due to local information governance and data protection regulations.

## Notes

**Steven M.A. Bow**, Visiting Research Fellow and Consultant in Public Health

**Peter Schröder-Bäck**, Professor for Ethics

**Dominic Norcliffe-Brown**, Senior Policy Advisor, Medical Ethics & Human Rights, Professional Policy & Activities

**James Wilson**, Professor of Philosophy

**Farhang Tahzib**, Chair of Ethics Committee and Public Health Physician
