# Supplementary material for: Moral distress and injury in the public health professional workforce during the COVID-19 pandemic
Source: J Public Health (Oxf). 2023 Mar 1;45(3):697–705. doi: 10.1093/pubmed/fdad010 (PMC10470334; doi:10.1093/pubmed/fdad010)
Supplement: FPH_Moral_distress_data_paper-JPH_v1-SUPP_fdad010 [file fph_moral_distress_data_paper-jph_v1-supp_fdad010.docx]

Moral distress and injury in the public health professional workforce during the COVID-19 pandemic

# Supplementary information

[Supplementary information 1](#_Toc117069608)

[Survey questions 2](#_Toc117069609)

[Supplementary Table 1. Detailed demographic and professional characteristics of respondents 9](#_Toc117069610)

[Supplementary Table 2. Associations between respondent characteristics and reported experience of moral distress during the COVID-19 pandemic 11](#_Toc117069611)

[Summary of logistic regression results 13](#_Toc117069612)

[Supplementary Table 3. Adjusted odds ratios for reported experience of moral distress for survey respondents 13](#_Toc117069613)

## Survey questions

**Question 1. Where have you primarily worked, since the beginning of the pandemic?**

- England
- Scotland
- Wales
- Northern Ireland
- Outside the UK (please specify)

**Question 2. For whom did you primarily work, since the beginning of the pandemic?**

- National public health agency (for example, Public Health England/UK Health Security Agency, Public Health Wales, Public Health Scotland, Public Health Agency N. Ireland)
- Office for Health Improvement and Disparities (UK)
- Local authority / local government
- University or academic institution
- NHS Trust or Health Board
- NHS England and NHS Improvement
- Department for Health and Social Care / other national government department
- Non-profit organisation/NGO
- Private practice
- Other (please specify)
- Not applicable

**Question 3. Which best describes your role, since the beginning of the pandemic?**

- Director of Public Health
- Consultant or Specialist in Public Health
- Public Health Academic
- Public Health Trainee / Specialty Registrar
- Public health manager
- Public health nurse or midwife
- Public Health Practitioner
- Retired
- Other (please specify)

**Question 4. How long have you been working in a professional public health role?**

- 5 years or less
- 6 to 10 years
- 11 to 15 years
- 16 to 20 years
- More than 20 years

**Question 5. Are you clinically trained?**

- Yes
- No

**Question 6. Since the start of the COVID-19 pandemic, in your professional work, have you had to do something that you thought was ethically problematic (morally wrong) – and experienced distress because of it?**

**For example, you may have been required to say or do something because of a law or policy that you thought was incorrect, and this caused you distress.**

- Yes
- No / not sure

You said that in the last year, in your professional work, you had to do something that you thought was ethically problematic (morally wrong) – and experienced distress because of it.

For example, you may have been required to say or do something because of a law or policy that you thought was incorrect, and this caused you distress.

**7. How often did you experience this kind of situation?**

- 1 – Rarely
- 2
- 3
- 4
- 5 - Very frequently

**Question 8. Did this occur more or less frequently than in the 12 months before the pandemic?**

- Less often
- About the same
- More often
- Not sure
- I was not working in public health during that period

Now, think about the single incident of this type that caused you the most distress...

**Question 9. Please indicate the level of distress you experienced because of this situation**

- 1. A little
- 2
- 3
- 4
- 5. Great extent

**Question 10. How long did you continue to experience distress because of this situation?**

- Less than a day
- Between a day and a week
- Between a week and month
- More than a month

**Question 11. Did the distress you experienced cause you to take time off from work, and/or seek therapeutic help?**

- Yes
- No

**Question 12. Please can you briefly describe the situation that caused you distress? Please avoid using real names or details of specific workplaces etc.**

**Question 13. What would help avoid or mitigate situations like this?**

**Question 14. Since the start of the COVID-19 pandemic, in your professional work, have you done something that you thought was the ethical (morally right) thing – and experienced distress because of it?**

**For example, you may have made a decision that you knew was right, but someone criticised you for it, or it made life more difficult for someone, and this caused you distress.**

- Yes
- No / not sure

You said that since the start of the COVID-19 pandemic, in your professional work, you did something that you thought was the ethical (morally right) thing – and experienced distress because of it.

For example, you may have made a decision that you knew was right, but someone criticised you for it, or it made life more difficult for someone, and this caused you distress.

**Question 15. How often did you experience this kind of situation?**

- 1 – Rarely
- 2
- 3
- 4
- 5 - Very frequently

**Question 16. Did this occur more or less frequently than in the 12 months before the pandemic?**

- Less often
- About the same
- More often
- Not sure
- I was not working in public health during that period

Now, think about the single incident of this type that caused you the most distress...

**Question 17. Please indicate the level of distress you experienced because of this situation**

- 1. A little
- 2
- 3
- 4
- 5. Great extent

**Question 18. How long did you continue to experience distress because of this situation?**

- Less than a day
- Between a day and a week
- Between a week and month
- More than a month

**Question 19. Did the distress you experienced cause you to take time off from work, and/or seek therapeutic help?**

- Yes
- No

**Question 20. Please can you briefly describe the situation that caused you distress? Please avoid using real names or details of specific workplaces etc.**

**Question 21. What would help avoid or mitigate situations like this?**

**Question 22. Since the start of the COVID-19 pandemic, in your professional work, have you had to do something where you were not sure what the ethical (morally right) thing to do was – and experienced distress because of it?**

**For example, you may have been unsure who to prioritise for a service, or whether or not to recommend a particular course of action, and this caused you distress.**

- Yes
- No / not sure

You said that, since the start of the COVID-19 pandemic, in your professional work, you had to do something where you were not sure what the ethical (or morally right) right thing to do was – and experienced distress because of it.

For example, you may have been unsure who to prioritise for a service, or whether or not to recommend a particular course of action, and this caused you distress.

**Question 23. How often did you experience this kind of situation?**

- 1 – Rarely
- 2
- 3
- 4
- 5 - Very frequently

**Question 24. Did this occur more or less frequently than in the 12 months before the pandemic?**

- Less often
- About the same
- More often
- Not sure
- I was not working in public health during that period

Now, think about the single incident of this type that caused you the most distress...

**Question 25. Please indicate the level of distress you experienced because of this situation**

- 1. A little
- 2
- 3
- 4
- 5. Great extent

**Question 26. How long did you continue to experience distress because of this situation?**

- Less than a day
- Between a day and a week
- Between a week and month
- More than a month

**Question 27. Did the distress you experienced cause you to take time off from work, and/or seek therapeutic help?**

- Yes
- No

**Question 28. Please can you briefly describe the situation that caused you distress? Please avoid using real names or details of specific workplaces etc.**

**Question 29. What would help avoid or mitigate situations like this?**

**Question 30. Since the start of the COVID-19 pandemic, in your professional work, has your colleague(s), or organisation, done something that you thought was ethically problematic (morally wrong) – and you experienced distress because of it?**

**For example, a manager may have made a decision that you thought would cause harm or be unfair, and this caused you distress.**

- Yes
- No / not sure

You said that in the last year, in your professional work, your colleague(s), or organisation, did something that you thought was ethically problematic (morally wrong) – and you experienced distress because of it.

For example, a manager may have made a decision that you thought would cause harm or be unfair, and this caused you distress.

**Question 31. How often did you experience this kind of situation?**

- 1 – Rarely
- 2
- 3
- 4
- 5 - Very frequently

**Question 32. Did this occur more or less frequently than in the 12 months before the pandemic?**

- Less often
- About the same
- More often
- Not sure
- I was not working in public health during that period

Now, think about the single incident of this type that caused you the most distress...

**Question 33. Please indicate the level of distress you experienced because of this situation**

- 1. A little
- 2
- 3
- 4
- 5. Great extent

**Question 34. How long did you continue to experience distress because of this situation?**

- Less than a day
- Between a day and a week
- Between a week and month
- More than a month

**Question 35. Did the distress you experienced cause you to take time off from work, and/or seek therapeutic help?**

- Yes
- No

**Question 36. Please can you briefly describe the situation that caused you distress? Please avoid using real names or details of specific workplaces etc.**

**Question 37. What would help avoid or mitigate situations like this?**

**Question 38. Have you heard of the term ‘moral distress’ before?**

- Yes
- No
- Not sure

**Question 39. Have you heard of the term ‘moral injury’ before?**

- Yes
- No
- Not sure

**Question 40. Have you had any specific training in ethics during your formal public health education/training (e.g. as part of a BSc, MSc, MPH)?**

- No
- Not sure
- Yes (please specify)

**Question 41. Have you had any specific training in ethics after your formal public health education (e.g. a continuing professional development session on ethics)?**

- No
- Not sure
- Yes (please specify)

**Question 42. Do you believe that more training in ethics would have helped you handle the situation(s) you describe earlier?**

- Yes
- No
- Not sure
- Not applicable

**Question 43. Please indicate the extent to which each of these corresponds to your own situation?**

|  | 0 - Never | 1 | 2 | 3 | 4 - Always |
| --- | --- | --- | --- | --- | --- |
| I can easily determine if a situation contains an ethical/moral dilemma |  |  |  |  |  |
| I know what principles, tools or frameworks to use to help me make a decision when confronted with ethical/moral issues |  |  |  |  |  |
| I think I am adequately prepared to face the ethical/moral issues related to my practice |  |  |  |  |  |
| I think that my colleagues are adequately prepared to face the ethical/moral issues related to their practice |  |  |  |  |  |

**Question 44. What is your gender?**

- Female
- Male
- Other (please specify)
- Prefer not to say

**Question 45. What is your age?**

- Under 18
- 18-24
- 25-34
- 35-44
- 45-54
- 55-64
- 65+
- Prefer not to say

**Question 46. Do you consider yourself to have a disability?**

- Yes
- No
- Prefer not to say

**Question 47. What is your religion?**

- Christian – Church of England/Ireland/Wales/Scotland
- Christian – Catholic
- Christian – Orthodox
- Other Christian, please describe below
- Buddhist
- Hindu
- Jewish
- Muslim
- Sikh
- No religion
- Other, please describe below
- Prefer not to say

Please describe (if indicated above)

**Question 48. Which ethnicity best describes you?**

- White - Scottish/English/Welsh/Northern Irish/British
- White - Irish
- White - Gypsy or Irish Traveller
- White - Any other White background, please describe below
- Mixed/Multiple - White and Black Caribbean
- Mixed/Multiple - White and Black African
- Mixed/Multiple - White and Asian
- Mixed/Multiple - Any other Mixed/Multiple ethnic background, please describe below
- Asian - Indian
- Asian - Pakistani
- Asian - Bangladeshi
- Asian - Chinese
- Asian - Any other Asian background, please describe below
- Black - African
- Black - Caribbean
- Black - Any other Black/African/Caribbean background, please describe below
- Other - Arab
- Other - Any other ethnic group, please describe below
- Prefer not to say

Please describe (if indicated above)

**Question 49. Please leave your email address here if you would you be willing to discuss these issues in an interview or focus group:**

**Question 50. Do you have any further comments about this survey, and/or public health ethics in general?**

## Supplementary Table 1. Detailed demographic and professional characteristics of respondents

| **Characteristic** | **N** | **P** |
| --- | --- | --- |
| **Gender** |  |  |
| Female | 308 | 49.0 |
| Male | 148 | 23.5 |
| Other (please specify) | 3 | 0.5 |
| Prefer not to say | 16 | 2.5 |
| Not stated | 154 | 24.5 |
| **Age** |  |  |
| Under 18 | 0 | 0.0 |
| 18-24 | 0 | 0.0 |
| 25-34 | 55 | 8.7 |
| 35-44 | 115 | 18.3 |
| 45-54 | 112 | 17.8 |
| 55-64 | 104 | 16.5 |
| 65+ | 67 | 10.7 |
| Prefer not to say | 22 | 3.5 |
| Not stated | 154 | 24.5 |
| **Disability** |  |  |
| Yes | 49 | 7.8 |
| No | 408 | 64.9 |
| Prefer not to say | 18 | 2.9 |
| Not stated | 154 | 24.5 |
| **Religion** |  |  |
| No religion | 231 | 36.7 |
| Christian – Church of England/Ireland/Wales/Scotland | 82 | 13.0 |
| Christian – Catholic | 37 | 5.9 |
| Other Christian, please describe below | 26 | 4.1 |
| Muslim | 14 | 2.2 |
| Other, please describe below | 9 | 1.4 |
| Hindu | 7 | 1.1 |
| Jewish | 7 | 1.1 |
| Buddhist | 4 | 0.6 |
| Christian – Orthodox | 3 | 0.5 |
| Sikh | 0 | 0.0 |
| Prefer not to say | 54 | 8.6 |
| Not stated | 155 | 24.6 |
| **Ethnicity** |  |  |
| White - Scottish/English/Welsh/Northern Irish/British | 320 | 50.9 |
| White - Any other White background, please describe below | 38 | 6.0 |
| White - Irish | 17 | 2.7 |
| Black - African | 16 | 2.5 |
| Asian - Indian | 12 | 1.9 |
| Asian - Any other Asian background, please describe below | 8 | 1.3 |
| Mixed/Multiple - White and Asian | 6 | 1.0 |
| Asian - Pakistani | 5 | 0.8 |
| Mixed/Multiple - White and Black African | 5 | 0.8 |
| Asian - Chinese | 4 | 0.6 |
| Other - Arab | 4 | 0.6 |
| Mixed/Multiple - White and Black Caribbean | 3 | 0.5 |
| Other - Any other ethnic group, please describe below | 3 | 0.5 |
| Asian - Bangladeshi | 1 | 0.2 |
| Black - Any other Black/African/Caribbean background, please describe below | 1 | 0.2 |
| Black - Caribbean | 1 | 0.2 |
| Mixed/Multiple - Any other Mixed/Multiple ethnic background, please describe below | 1 | 0.2 |
| White - Gypsy or Irish Traveller | 0 | 0.0 |
| Prefer not to say | 27 | 4.3 |
| Not stated | 157 | 25.0 |
| **Country** |  |  |
| England | 479 | 76.2 |
| Outside the UK (please specify) | 63 | 10.0 |
| Scotland | 42 | 6.7 |
| Wales | 29 | 4.6 |
| Northern Ireland | 16 | 2.5 |
| Not stated | 0 | 0.0 |
| **Employer** |  |  |
| Local authority / local government | 206 | 32.8 |
| National public health agency (for example, Public Health England/UK Health Security Agency, Public Health Wales, Public Health Scotland, Public Health Agency N. Ireland) | 140 | 22.3 |
| University or academic institution | 76 | 12.1 |
| Other (please specify) | 64 | 10.2 |
| NHS Trust or Health Board | 53 | 8.4 |
| Department for Health and Social Care / other national government department | 22 | 3.5 |
| NHS England and NHS Improvement | 14 | 2.2 |
| Non-profit organisation/NGO | 12 | 1.9 |
| Private practice | 10 | 1.6 |
| Office for Health Improvement and Disparities (UK) | 5 | 0.8 |
| Not applicable | 26 | 4.1 |
| Not stated | 1 | 0.2 |
| **Job role** |  |  |
| Consultant or Specialist in Public Health | 207 | 32.9 |
| Public Health Trainee / Specialty Registrar | 132 | 21.0 |
| Other (please specify) | 84 | 13.4 |
| Public Health Academic | 59 | 9.4 |
| Director of Public Health | 49 | 7.8 |
| Retired | 39 | 6.2 |
| Public Health Practitioner | 30 | 4.8 |
| Public health manager | 23 | 3.7 |
| Public health nurse or midwife | 4 | 0.6 |
| Not stated | 2 | 0.3 |
| **Duration of professional public health role** |  |  |
| 5 years or less | 147 | 23.4 |
| 6 to 10 years | 112 | 17.8 |
| 11 to 15 years | 80 | 12.7 |
| 16 to 20 years | 78 | 12.4 |
| More than 20 years | 212 | 33.7 |
| Not stated | 0 | 0.0 |
| **Clinically trained** |  |  |
| Yes | 369 | 58.7 |
| No | 259 | 41.2 |
| Not stated | 1 | 0.2 |
| **Heard of ‘moral distress’** |  |  |
| Yes | 288 | 45.8 |
| No | 149 | 23.7 |
| Not sure | 41 | 6.5 |
| Not stated | 151 | 24.0 |
| **Heard of ‘moral injury’** |  |  |
| Yes | 238 | 37.8 |
| No | 202 | 32.1 |
| Not sure | 37 | 5.9 |
| Not stated | 152 | 24.2 |
| **Received ethical training *during* formal public health education** |  |  |
| Yes | 225 | 35.8 |
| No | 173 | 27.5 |
| Not sure | 79 | 12.6 |
| Not stated | 152 | 24.2 |
| **Received ethical training *since* formal public health education** |  |  |
| Yes | 164 | 26.1 |
| No | 259 | 41.2 |
| Not sure | 53 | 8.4 |
| Not stated | 153 | 24.3 |
| **Believed more ethical training would have helped** |  |  |
| Yes | 150 | 23.8 |
| No | 87 | 13.8 |
| Not sure | 165 | 26.2 |
| Not applicable | 72 | 11.4 |
| Not stated | 155 | 24.6 |

## Supplementary Table 2. Associations between respondent characteristics and reported experience of moral distress during the COVID-19 pandemic

|  |  | **Moral distress due to own actions** | | | |  | **Moral distress due to actions of colleague/ organisation** | | | |
| --- | --- | --- | --- | --- | --- | --- | --- | --- | --- | --- |
|  |  | **No** | **Yes** |  |  |  | **No** | **Yes** |  |  |
| **Respondent characteristics** |  | N (%) | N (%) | Chi^2^ | p-value |  | N (%) | N (%) | Chi^2^ | p-value |
| **Gender** |  |  |  |  |  |  |  |  |  |  |
| Female |  | 109 (35) | 199 (65) |  |  |  | 207 (67) | 101 (33) |  |  |
| Male |  | 60 (41) | 88 (59) | 1·14 | 0·29 |  | 103 (70) | 45 (30) | 0·26 | 0·61 |
| **Age** |  |  |  |  |  |  |  |  |  |  |
| 25-34 |  | 17 (31) | 38 (69) |  |  |  | 41 (75) | 14 (25) |  |  |
| 35-44 |  | 25 (22) | 90 (78) |  |  |  | 74 (64) | 41 (36) |  |  |
| 45-54 |  | 35 (31) | 77 (69) |  |  |  | 69 (62) | 43 (38) |  |  |
| 55-64 |  | 42 (40) | 62 (60) |  |  |  | 68 (65) | 36 (35) |  |  |
| 65+ |  | 46 (69) | 21 (31) | 43·49 | **<0·01** |  | 57 (85) | 10 (15) | 13·23 | **0·01** |
| **Disability** |  |  |  |  |  |  |  |  |  |  |
| Yes |  | 16 (33) | 33 (67) |  |  |  | 25 (51) | 24 (49) |  |  |
| No |  | 151 (37) | 257 (63) | 0·36 | 0·55 |  | 284 (70) | 124 (30) | 6·90 | **<0·01** |
| **Religion** |  |  |  |  |  |  |  |  |  |  |
| No religion |  | 81 (35) | 150 (65) |  |  |  | 154 (67) | 77 (33) |  |  |
| Christian |  | 55 (37) | 93 (63) |  |  |  | 103 (70) | 45 (30) |  |  |
| Other religion |  | 20 (49) | 21 (51) | 2·81 | 0·25 |  | 31 (76) | 10 (24) | 1·40 | 0·50 |
| **Ethnicity** |  |  |  |  |  |  |  |  |  |  |
| White |  | 134 (36) | 241 (64) |  |  |  | 254 (68) | 121 (32) |  |  |
| Other |  | 31 (44) | 39 (56) | 1·85 | 0·17 |  | 50 (71) | 20 (29) | 0·37 | 0·54 |
| **Country** |  |  |  |  |  |  |  |  |  |  |
| England |  | 168 (35) | 311 (65) |  |  |  | 355 (74) | 124 (26) |  |  |
| UK devolved administration |  | 24 (28) | 63 (72) |  |  |  | 65 (75) | 22 (25) |  |  |
| Outside the UK |  | 32 (51) | 31 (49) | 8·84 | **0·01** |  | 46 (73) | 17 (27) | 0·06 | 0·97 |
| **Employer** |  |  |  |  |  |  |  |  |  |  |
| Local government |  | 57 (28) | 149 (72) |  |  |  | 155 (75) | 51 (25) |  |  |
| National government / agency |  | 58 (32) | 123 (68) |  |  |  | 129 (71) | 52 (29) |  |  |
| Academic institution |  | 38 (50) | 38 (50) |  |  |  | 52 (68) | 24 (32) |  |  |
| Healthcare |  | 12 (23) | 41 (77) |  |  |  | 40 (75) | 13 (25) |  |  |
| Other |  | 35 (41) | 51 (59) | 17·46 | **<0·01** |  | 64 (74) | 22 (26) | 1·86 | 0·76 |
| **Job role** |  |  |  |  |  |  |  |  |  |  |
| Consultant / Specialist |  | 58 (28) | 149 (72) |  |  |  | 142 (69) | 65 (31) |  |  |
| Public Health Trainee |  | 39 (30) | 93 (70) |  |  |  | 103 (78) | 29 (22) |  |  |
| Public Health Academic |  | 31 (53) | 28 (47) |  |  |  | 40 (68) | 19 (32) |  |  |
| Director of Public Health |  | 8 (16) | 41 (84) |  |  |  | 33 (67) | 16 (33) |  |  |
| Retired |  | 30 (77) | 9 (23) |  |  |  | 35 (90) | 4 (10) |  |  |
| Other |  | 57 (40) | 84 (60) | 53·13 | **<0·01** |  | 111 (79) | 30 (21) | - | **0·02*** |
| **Public health career length** |  |  |  |  |  |  |  |  |  |  |
| 5 years or less |  | 47 (32) | 100 (68) |  |  |  | 112 (76) | 35 (24) |  |  |
| 6 to 10 years |  | 23 (21) | 89 (79) |  |  |  | 83 (74) | 29 (26) |  |  |
| 11 to 15 years |  | 22 (28) | 58 (73) |  |  |  | 60 (75) | 20 (25) |  |  |
| 16 to 20 years |  | 22 (28) | 56 (72) |  |  |  | 54 (69) | 24 (31) |  |  |
| More than 20 years |  | 110 (52) | 102 (48) | 40·60 | **<0·01** |  | 157 (74) | 55 (26) | 1·33 | 0·86 |
| **Clinically trained** |  |  |  |  |  |  |  |  |  |  |
| Yes |  | 143 (39) | 226 (61) |  |  |  | 272 (74) | 97 (26) |  |  |
| No |  | 81 (31) | 178 (69) | 3·71 | 0·05 |  | 193 (75) | 66 (25) | 0·05 | 0·82 |
| **Ethical training received during formal education** |  |  |  |  |  |  |  |  |  |  |
| Yes |  | 72 (32) | 153 (68) |  |  |  | 144 (64) | 81 (36) |  |  |
| No/not sure |  | 102 (40) | 150 (60) | 3·69 | 0·06 |  | 177 (70) | 75 (30) | 2·10 | 0·15 |
| **Ethical training received since formal education** |  |  |  |  |  |  |  |  |  |  |
| Yes |  | 77 (47) | 87 (53) |  |  |  | 114 (70) | 50 (30) |  |  |
| No/not sure |  | 98 (31) | 214 (69) | 11·17 | **<0·01** |  | 207 (66) | 105 (34) | 0·49 | 0·48 |

* P-value derived from Fisher’s exact test due to small cell numbers

## Summary of logistic regression results

Increasing age was inversely associated with the odds of experiencing moral distress due to one’s own actions, with a 68%, 84% and 90% decrease in the odds of experiencing moral distress for those in the 45-54, 55-64 and 65+ year age groups, respectively, compared to the 25-34 reference group (adjusted ORs=0·32, 0·16, 0·10; 95%CIs=0·11-0·94, 0·05-0·55, 0·02-0·40). There was a 54% decrease in the odds of experiencing moral distress due to one’s own actions for those of other ethnic groups compared to those in the white ethnic group (adjusted OR=0·46; 95%CI=0·21-0·97). There was a 61% and 77% decrease in the odds of experiencing moral distress due to one’s own actions for public health trainees and “other” job roles compared to the consultant/specialist reference group (adjusted ORs=0·39, 0.23; 95%CIs=0·19-0·79, 0·09-0·56). There was no clear linear trend in association between public health career length and the odds of experiencing moral distress due to one’s own actions, however those in the 6-10 years group had 193% greater odds of experiencing moral distress due to one’s own actions compared to those whose career duration was 5 years or fewer (adjusted OR=2·93; 95%CI=1·26-6·81). Those who had received ethical training since the end of their formal education had 48% lower odds of experiencing moral distress due to one’s own actions (adjusted OR=0·52; 95%CI=0·31-0·87). For moral distress due to the actions of colleagues or an organisation, the odds of experiencing such distress were 143% higher for those reporting a disability (adjusted OR=2·43; 95%CI=1·15-5·14).

## Supplementary Table 3. Adjusted odds ratios for reported experience of moral distress for survey respondents

|  |  |  | **Moral distress due to own actions** | |  | **Moral distress due to actions of colleague/ organisation** | |
| --- | --- | --- | --- | --- | --- | --- | --- |
| **Respondent characteristics** | |  | **OR** | **95% CIs** |  | **OR** | **95% CIs** |
| **Gender** | Female |  | 1·00 |  |  | 1·00 |  |
|  | Male |  | 1·04 | 0·60-1·82 |  | 0·95 | 0·55-1·62 |
| **Age** | 25-34 |  | 1·00 |  |  | 1·00 |  |
|  | 35-44 |  | 0·80 | 0·32-1·98 |  | 1·35 | 0·55-3·32 |
|  | 45-54 |  | **0·32** | **0·11-0·94** |  | 1·18 | 0·42-3·31 |
|  | 55-64 |  | **0·16** | **0·05-0·55** |  | 0·87 | 0·27-2·87 |
|  | 65+ |  | **0·10** | **0·02-0·40** |  | 0·45 | 0·11-1·88 |
| **Disability** | No |  | 1·00 |  |  | 1·00 |  |
|  | Yes |  | 1·31 | 0·57-3·00 |  | **2·43** | **1·15-5·14** |
| **Religion** | No religion |  | 1·00 |  |  | 1·00 |  |
|  | Christian |  | 0·84 | 0·49-1·43 |  | 0·75 | 0·44-1·27 |
|  | Other religion |  | 0·85 | 0·36-2·01 |  | 0·59 | 0·24-1·46 |
| **Ethnicity** | White |  | 1·00 |  |  | 1·00 |  |
|  | Other |  | **0·46** | **0·21-0·97** |  | 1·01 | 0·47-2·18 |
| **Country** | England |  | 1·00 |  |  | 1·00 |  |
|  | UK devolved administration |  | 1·21 | 0·54-2·73 |  | 1·35 | 0·62-2·95 |
|  | Other country |  | 0·92 | 0·38-2·24 |  | 1·60 | 0·67-3·84 |
| **Employer** | Academic institution |  | 1·00 |  |  | 1·00 |  |
|  | National government / agency |  | 0·61 | 0·19-1·92 |  | 0·66 | 0·20-2·16 |
|  | Local government |  | 0·76 | 0·24-2·44 |  | 0·56 | 0·17-1·88 |
|  | Healthcare |  | 1·73 | 0·40-7·49 |  | 0·70 | 0·17-2·84 |
|  | Other |  | 1·42 | 0·44-4·53 |  | 0·73 | 0·22-2·46 |
| **Job role** | Consultant / Specialist |  | 1·00 |  |  | 1·00 |  |
|  | Director of Public Health |  | 2·46 | 0·85-7·09 |  | 1·25 | 0·54-2·88 |
|  | Other |  | **0·39** | **0·19-0·79** |  | 0·56 | 0·28-1·13 |
|  | Public Health Academic |  | 0·34 | 0·09-1·25 |  | 0·68 | 0·19-2·49 |
|  | Public Health Trainee |  | **0·23** | **0·09-0·56** |  | 0·55 | 0·25-1·22 |
|  | Retired |  | 0·46 | 0·12-1·75 |  | 1·04 | 0·23-4·61 |
| **Public health career length** | 5 years or less |  | 1·00 |  |  | 1·00 |  |
|  | 6 to 10 years |  | **2·93** | **1·26-6·81** |  | 0·80 | 0·38-1·70 |
|  | 11 to 15 years |  | 1·75 | 0·65-4·65 |  | 0·99 | 0·40-2·44 |
|  | 16 to 20 years |  | 2·99 | 0·96-9·33 |  | 1·29 | 0·47-3·52 |
|  | More than 20 years |  | 1·50 | 0·55-4·14 |  | 0·94 | 0·35-2·53 |
| **Clinically trained** | No |  | 1·00 |  |  | 1·00 |  |
|  | Yes |  | 1·20 | 0·71-2·03 |  | 1·18 | 0·72-1·95 |
| **Ethical training received during formal education** | No/not sure |  | 1·00 |  |  | 1·00 |  |
|  | Yes |  | 1·13 | 0·68-1·85 |  | 1·25 | 0·78-2·01 |
| **Ethical training received since formal education** | No/not sure |  | 1·00 |  |  | 1·00 |  |
|  | Yes |  | **0·52** | **0·31-0·87** |  | 0·76 | 0·45-1·28 |
| **Constant** |  |  | **9·20** | **1·83-46·20** |  | 0·79 | 0·16-3·83 |
